# Embryonic stem cells go from bench to bedside for Parkinson’s disease

**DOI:** 10.1016/j.xcrm.2021.100251

**Published:** 2021-04-20

**Authors:** Clare L. Parish, Lachlan H. Thompson

**Affiliations:** 1 The Florey Institute of Neuroscience and Mental Health, Parkville, VIC, Australia

## Abstract

Stem-cell-derived transplants may soon be a promising treatment option for Parkinson’s disease. In preparation for clinical trial, Piao et al.^1^ report on generating a clinical-grade dopaminergic progenitor cell product and its rigorous testing to ensure safety and efficacy.

## Main Text

Progressive degeneration of midbrain dopamine neurons underpins many pathophysiological symptoms of Parkinson’s disease (PD). While mainstay dopamine pharmacotherapies provide good symptomatic relief in early disease stages, they present side effects and fail to address neuronal loss. In contrast, targeted cell grafts can replace dopamine neurons. Supporting this approach, clinical trials using human fetal tissue have demonstrated graft survival, integration, and sustained symptomatic relief in many patients. However, lack of standardization, ethical concerns, and tissue availability highlight the need for an alternative donor source. Here, human pluripotent stem cells (hPSCs), including induced (IPSCs) and embryonic (ESCs), present clear advantages—being highly expandable and amenable to standardized fate restriction under defined conditions such that large-scale production of a donor cell product can be achieved. Progress in this area has led to a recent clinical trial using IPSCs in Japan,^2^ and a single patient in the United States receiving an autologous IPSC-derived neural graft generated from his own cells.^3^

Piao et al. recently reported on the generation of the first ESC-derived dopamine neuron cell product for the treatment of PD.^1^ This team (led by Tabar, Studer, and Tomishima) has long been a leading force in the development of procedures to produce dopamine neurons from hPSCs. Achieving initial success by combining dual SMAD inhibition to control neural induction and ventralized floor specification to generate ventral midbrain dopamine neurons,^4^ subsequent efforts to adapt the protocol to clinically compatible conditions resulted in near complete loss of the dopamine neurons. To address this, in a companion paper, the team recently established a revised protocol involving biphasic modulation of WNT signaling to control the rostrocaudal specification of the progenitors.^5^ This has enabled the team to now embark on the first clinical trial using human ESC-derived dopamine progenitors.

Working with hESC line WA09 (originally generated at the University of Wisconsin), Dr. Tabar and her team performed four large-scale manufacturing and cryopreservation rounds to generate clinical-grade progenitors, named MSK-DA01. The cell product was scrutinized for quality control to verify ventral midbrain specification, negligible presence of off-target, or immature neural stem cell populations as well as absence of undifferentiated ESCs.

Prior to clinical translation regulatory approval by the Food and Drug Administration (FDA) required rigorous testing of the cell product to ensure safety, reproducibility, and efficacy. Impressively, Piao et al. performed grafts into more than 350 immune-compromised animals to address these requirements. A series of toxicity, biodistribution, and tumorigenicity studies, conducted under good laboratory manufacturing practice (GLP) guidelines, confirmed that grafts of the MSK-DA01 cells showed no adverse effects. Assessments showed no signs of cytotoxicity or abnormal proliferation, while detection of human DNA outside the brain (indicative of cell trafficking) was rare and likely reflected a technical injection issue.

Acute grafting studies confirmed graft survival and presence of dopamine neurons at 3 weeks. Subsequent long-term efficacy studies demonstrated the capacity of these MSK-DA01 cells to fully reverse motor symptoms in an induced hemiparkinson rat model. Histological assessment at 8 months verified the presence of dopamine neurons (estimated at 10% of total cells within the graft), the negligible presence of proliferative cells, and the absence of serotonergic neurons—the latter being an unwanted off-target population that can contribute to dyskinesias.

**Figure 1:**
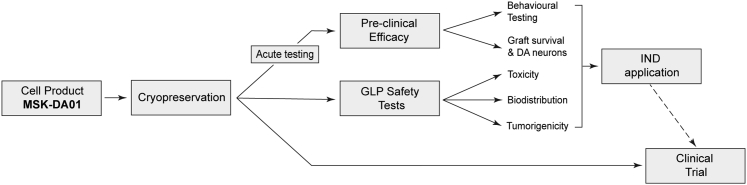
Workflow for moving ESCs into the clinic for PD Pipeline for the development, pre-clinical testing and regulatory approval of an hESC-derived midbrain dopamine progenitor product, MSK-DA01, suitable for grafting in PD patients.

The team has now successfully executed the full pipeline of work from optimization of the protocol (including GLP standardization and current good manufacturing practice (cGMP) compliance), establishment of cryopreservation procedures, and necessary safety and efficacy testing (Figure 1). Harmonization of technically demanding aspects of the basic science involved in directed differentiation and transplantation of DA progenitors with challenges imposed by scaling, regulatory compliance, and implementation in a clinical environment is an outstanding achievement. After this rigorous journey, and having recently received FDA approval for their IND (investigational new drug) submission, they are now poised to embark on the first phase I clinical trial using ESC-derived neural progenitors for PD. In this new contribution, Piao et al. discuss trial design, considerate of patient selection, optimal surgical approach, cell dosage, and immune suppression regime, as well as primary and secondary endpoints that will address both safety and efficacy.

While the preclinical testing at present is onerous and varies depending on national regulatory agencies, this will likely change as the field learns more from on-going and proposed clinical trials. It is plausible that some of the current required steps may be disregarded, or become more streamlined, while new elements may also be implemented. For these reasons, it will be critically that teams around the world work together to share their trial findings. Having learnt from human fetal tissue trials in PD patients in the 1990s (where outcomes showed promise but were highly variable), those embarking on new trials have elected to band together in a collegial manner forming the consortium, “GForce-PD” (http://www.gforce-pd.com), in order to achieve uniform success toward the common goal of advancing hPSC-derived neural grafts into the clinic for PD.^6^

It remains early days in the use PSCs for clinical application and the outcome of these trials will be critical for moving stem cell grafting more prominently into clinical practice for the treatment of PD. There are of course additional areas that warrant ongoing attention as scientific knowledge progresses that may prove to refine the approach and further optimize clinical outcomes. In particular, strategies to improve graft purity and limit the expansion of off-target/non-dopaminergic populations through the pre-selection of correctly specified progenitors prior to grafting^7^ or elimination of unwanted populations through suicide-based approaches after implantation (C.L.P., unpublished data). Added to this is the consideration that additional trophic cues may enhance graft survival, fate acquisition, and plasticity as recently demonstrated,^8^ or whether homotopic transplants, into the site of cell loss, is feasible in the human brain. Finally, while the consensus surrounding current and proposed trials is that a period of immunosuppression is needed after grafting, whether engineered universal donor cells can circumvent this for neural grafting remains to be seen.^9^

In summary, it will indeed be an exciting few years ahead as we await the outcome of the clinical trial of Dr. Tabar and her team, as well as other groups associated with GForce-PD, collectively aspiring to develop alternative, cell-based therapies for the treatment of PD.^6^

## References

[1] Piao J., Zabierowski S., Dubose B.N., Hill E.J., Navare M., Claros N., Rosen S., Ramnarine K., Horn C., Fredrickson C. (2021). Preclinical Efficacy and Safety of a Human Embryonic Stem Cell-Derived Midbrain Dopamine Progenitor Product, MSK-DA01. Cell stem cell..

[2] Doi D., Magotani H., Kikuchi T., Ikeda M., Hiramatsu S., Yoshida K., Amano N., Nomura M., Umekage M., Morizane A., Takahashi J. (2020). Pre-clinical study of induced pluripotent stem cell-derived dopaminergic progenitor cells for Parkinson’s disease. Nat. Commun..

[3] Schweitzer J.S., Song B., Herrington T.M., Park T.Y., Lee N., Ko S., Jeon J., Cha Y., Kim K., Li Q. (2020). Personalized iPSC-Derived Dopamine Progenitor Cells for Parkinson’s Disease. N. Engl. J. Med..

[4] Kriks S., Shim J.W., Piao J., Ganat Y.M., Wakeman D.R., Xie Z., Carrillo-Reid L., Auyeung G., Antonacci C., Buch A. (2011). Dopamine neurons derived from human ES cells efficiently engraft in animal models of Parkinson’s disease. Nature.

[5] Kim T.W., Piao J., Koo S.Y., Kriks S., Chung S.Y., Betel D., Socci N.D., Choi S.J., Zabierowski S., Dubose B.W. (2021). Biphasic Activation of WNT Signaling Facilitates the Derivation of Midbrain Dopamine Neurons from hESCs for Translational Use. Cell stem cell..

[6] Barker R.A., Parmar M., Studer L., Takahashi J. (2017). Human Trials of Stem Cell-Derived Dopamine Neurons for Parkinson’s Disease: Dawn of a New Era. Cell Stem Cell.

[7] Samata B., Doi D., Nishimura K., Kikuchi T., Watanabe A., Sakamoto Y., Kakuta J., Ono Y., Takahashi J. (2016). Purification of functional human ES and iPSC-derived midbrain dopaminergic progenitors using LRTM1. Nat. Commun..

[8] Gantner C.W., de Luzy I.R., Kauhausen J.A., Moriarty N., Niclis J.C., Bye C.R., Penna V., Hunt C.P.J., Ermine C.M., Pouton C.W. (2020). Viral Delivery of GDNF Promotes Functional Integration of Human Stem Cell Grafts in Parkinson’s Disease. Cell stem cell..

[9] Lanza R., Russell D.W., Nagy A. (2019). Engineering universal cells that evade immune detection. Nat. Rev. Immunol..

